# Supplementary material for: Mechanics of pandemics
Source: eClinicalMedicine. 2026 Jul 31;98:104101. doi: 10.1016/j.eclinm.2026.104101 (PMC13453475; doi:10.1016/j.eclinm.2026.104101)
Supplement: Supplementary file 1 [file mmc1.docx]

**Supplementary Material of “Mechanics of Pandemics”**

**Appendix A: System-level monitoring and the limits of outbreak-based prevention**

This section expands mechanism M1 by clarifying why prevention cannot rely only on detecting outbreaks after human cases have already appeared. The mechanism is based on a mismatch between the timescale on which emergence risk accumulates and the timescale on which outbreaks are usually detected. Spillover and early transmission may be preceded by changes in ecological, epidemiological, behavioural, or environmental conditions, but public-health systems often respond only once human cases are observed and reported. At that stage, the system may already have moved from a low-risk configuration into a high-risk state in which sustained transmission, stochastic amplification, or rapid exponential growth becomes more likely.

This mechanism connects directly to the early-growth mechanism described in Section F. Once sustained human-to-human transmission is established, even short delays in detection or response can produce large differences in cumulative incidence. It also interacts with stochasticity and superspreading: near the epidemic threshold, small transmission clusters may either disappear by chance or expand rapidly before routine casebased surveillance detects a clear signal. Thus, prevention based only on observed outbreaks responds to a late manifestation of a broader system-level transition.

The practical implication is that prevention requires monitoring the broader system in which emergence becomes possible. In high-resource settings, this may include wastewater surveillance, genomic monitoring, sentinel surveillance in livestock and wildlife, vector surveillance, environmental monitoring, and integrated data platforms. In lower-resource settings, the same principle may be implemented through community-based surveillance, syndromic reporting, animal-health networks, targeted ecological monitoring, and strengthened communication between human-health, veterinary, and environmental sectors. The mechanism is therefore not tied to a single technology, but to the integration of multiple weak signals across the One Health system.

A key limitation is that upstream warning signals are often noisy, incomplete, and probabilistic. Detecting a change in system risk does not automatically identify which intervention is justified, when it should be triggered, or how costly false alarms may be. This creates a communication challenge: authorities must be able to explain why action may be needed before a visible outbreak occurs. If early warnings are poorly communicated, repeated false alarms may undermine trust and reduce compliance. Thus, the effectiveness of this mechanism depends not only on surveillance capacity, but also on data integration, institutional coordination, public trust, and transparent risk communication.

**Appendix B: Two-intervention algebra with absolute overlap**

This section provides an extended mathematical framework for mechanism 2.

We consider an SIR-like model with baseline transmission rate $\beta_{0}$ (no NPIs) and recovery rate $\gamma$.

Let two NPIs be indexed by 1, 2. Each NPI, when applied alone, removes a fraction $\varepsilon\in[0,1]$ of risky contacts.

Define $\phi\in[0,1]$ as the *absolute* fraction of contacts removed by *both* NPIs, i.e., the measure of the intersection:

$$\phi= |A\cap B|,$$

where $|A|=|B|=\varepsilon$ are the (fractional) contact sets removed by NPI 1 and NPI 2 respectively.

**Union (combined) reduction**

The total fraction of contacts removed by the union of the two NPIs equals

$$r_{\mathrm{union}}=\left| A\cup B \right|= \left| A \right|+\left| B \right|-\left| A\cap B \right|= 2\varepsilon-\phi. (1)$$

The transmission rate under both NPIs is therefore

$$\beta=\beta_{0} \left[ 1-r_{\mathrm{union}} \right]=\beta_{0}\left[ 1-2\varepsilon\right]. (2)$$

Consequently, the instantaneous effective reproduction number is

$$R_{\mathrm{eff}}(t)=\frac{\beta\left( t \right)}{\gamma}\frac{S\left( t \right)}{N}= R_{0}\frac{S\left( t \right)}{N}\left[ 1-2\varepsilon+\phi\right], (3)$$

with $R_{0}=\frac{\beta_{0}}{\gamma}$

**Relation to multiplicative (independence) model**

The standard multiplicative (probabilistic independence) model yields the joint retention factor $\left( 1-\varepsilon\right)^{2}$ , hence the combined fractional reduction

$$r_{\mathrm{mult}} = 1-\left( 1-\varepsilon\right)^{2} = 2\varepsilon-\varepsilon^{2}. (4)$$

Independence corresponds to the special overlap value $\phi=\varepsilon^{2}$, because substituting $\phi=\varepsilon^{2}$ into eq. (1) recovers eq. (4) (under independence, $\varepsilon^{2}$ is the probability that a contact is a member of contact sets $A$ and $B$ simultaneously). The difference between the union and multiplicative reductions is

$$r_{\mathrm{union}}-r_{\mathrm{mult}}=\varepsilon^{2} - \phi$$

Thus

- $\phi>\varepsilon^{2}$ implies $r_{\mathrm{union}}<r_{\mathrm{mult}}$ (greater overlap than independence; *redundancy*),
- $\phi=\varepsilon^{2}$ implies multiplicative/independent behaviour,
- $\phi<\varepsilon^{2}$ implies $r_{\mathrm{union}}>r_{\mathrm{mult}}$ (less overlap than independence; *synergy*).

Note that for the intersection it must hold that $0\leq\phi\leq\varepsilon$. Moreover, the union cannot exceed the whole contact set, so $r_{\mathrm{union}}\leq1$, which implies $\phi\geq2\varepsilon- 1$.

One may also choose to reparameterise departures from independence by the relative deviation,

$$\varphi=\frac{\phi-\varepsilon^{2}}{\varepsilon^{2}},$$

so that $\varphi=0$ is independence, $\varphi>0$ is excess overlap (sub-additivity) and $\varphi<0$ is deficit overlap (super-additivity). Estimation of $\phi$ requires data with variation in joint policy deployment; co-occurrence of NPIs limits identifiability and motivates use of staggered rollouts or instrumental variation.

**Appendix C: Practical challenges in evaluating NPI interactions**

The theoretical results on NPI redundancy and synergy outlined in mechanism M2 have important implications for the design, synthesis, and interpretation of empirical research on intervention effectiveness. While the algebra of NPI interactions can be formalized cleanly in a mathematical framework, the practical evaluation of the effectiveness of combined interventions is challenging and raises several issues, which we address below.

An immediate practical consequence is that effectiveness estimates for individual NPIs cannot be directly compared across studies that differ in the set of co-deployed measures. Because the marginal effect of any single NPI depends on the existing portfolio of controls [1,2], estimates of the effect of a given intervention, such as mask mandates, will generally differ depending on which other measures are simultaneously in place, for example, school closures. Direct comparability would require all studies to consider the same set of NPIs being studied in conjunction, a condition that is hardly ever satisfied in practice, given the heterogeneity of pandemic policy responses across countries and time periods [3]. Researchers synthesizing evidence on NPIs drawn from different studies must therefore explicitly account for this co-intervention dependence, and accurately and proactively communicate potential uncertainties that arise from aggregating estimates across heterogeneous policy environments. This comparability problem reflects the deeper structural issue that NPIs do not have fixed, context-independent effects—a point that directly motivates the need for careful study and model design.

Addressing the comparability problem requires moving away from the treatment of NPIs as independent clinical interventions whose effects can be simply added or pooled. Rather, they are highly endogenous, deeply correlated disruptions to a complex adaptive social system [4–7]. At the model-design stage, defaulting to a simple log-linear reduction on transmission of the form $R_{t} = R_{0} -\sum_{i} \alpha_{i}X_{i}$, implicitly assumes independence between measures and will misrepresent the redundancy structure described in Appendix C. More appropriate functional forms should allow for multiplicative interactions, ceiling effects, and regularized interaction terms that reflect diminishing returns as the portfolio of active measures grows [1,2]. Including high-frequency behavioral proxies alongside NPI indicator variables—such as aggregate mobile phone mobility data, consumer transaction indicators, or public transport usage—can help to assess behavioral responses [8,9]. At the cross-study synthesis stage, NPI effect sizes should never be pooled across studies without adjusting for the co-intervention background, for instance, as provided by government response trackers in the case of COVID-19 [3]. In meta-analyses, stratification or weighting should account for the strength of the causal identification strategy, not merely statistical sample size. Even with these refinements in place, however, a more fundamental modeling challenge remains.

At a deeper level, the challenge epidemiological modelers face here is a version of the Lucas Critique from macroeconomics [10], which states that historical behavioral relationships cannot be reliably used to predict the effects of a policy intervention because policy changes themselves change behavior. While traditional compartmental models are well-suited to represent biological transmission mechanisms, their parameters are typically estimated under behavioral regimes that may not persist under policy innovations or voluntary behavioral adaptation [11,12]. As a result, a model calibrated during a period of voluntary distancing may misrepresent the counterfactual scenario with no formal NPIs, since the behavioral responses to perceived risk are themselves endogenous to the policy environment—the very feedback mechanism discussed in mechanism 10. To address this in practice, strategies from economics to identify causal effects and avoid structural biases offer increasingly promising future research avenues. These include structural equation modeling to isolate unexpected policy shocks via impulse response functions; integration of behavioral and game-theoretic feedback loops directly into transmission models [13,14]; and the application of the causal inference toolkit from econometrics, such as staggered difference-in-differences designs [15], and regression discontinuity; and synthetic control methods [16]. Taken together, these approaches would allow researchers to better separate the causal effect of formal interventions from the confounding influence of endogenous behavioral change, ultimately producing more robust and policy-relevant estimates of NPI effectiveness—and bringing empirical practice closer to the theoretical framework laid out in Appendix C.

**Appendix D: The algebra of the quadratic effect in the SIR model**

This section provides an extended mathematical framework for mechanism 3.

In the classical SIR model, the number of new infections $\Delta I_{+}$ is proportional to the positive term in the derivative of the infectious compartment ($\beta IS$). Explicitly, if $S$ denotes the number of susceptible, $I$ the number of infectious individuals, and $\beta$ the transmission rate, the dynamics of the infectious compartment are given by

$$\frac{dI}{dt}=\beta IS-\gamma I,$$

where $\gamma$is the recovery rate. The number of new infections is therefore proportional to the product of the number of susceptible and the number of infectious individuals. Reducing the attendance of an event to a fraction $\kappa$ of the original number of participants reduces on average both the number of susceptibles and the number of infectious individuals present at the event. Mathematically, the new number of infections is reduced quadratically

$$\Delta I_{+}\left( \kappa\right)\sim\left( \kappa I \right)\cdot\left( \kappa S \right)= \kappa^{2}IS$$

If we consider $N$ events that all have the same fractions of infectious and susceptible people, reducing the attendance in each event to a fraction $\kappa$ gives again the quadratic reduction of new cases

$$\Delta I_{+}\left( \kappa\right)\sim\sum_{\mathrm{event}=1}^{N} \left( \kappa I_{\mathrm{event}} \right)\cdot\left( \kappa S_{\mathrm{event}} \right)= \kappa^{2}\sum_{\mathrm{event}=1}^{N} I_{\mathrm{event}}S_{\mathrm{event}}=\kappa^{2}\Delta I_{+}$$

In contrast, reducing the number of events to $\kappa N$ while keeping full attendance at each event yields only a linear reduction

$$\Delta I_{+}\left( \kappa\right)\sim\sum_{\mathrm{event}=1}^{\kappa N} I_{\mathrm{event}}S_{\mathrm{event}}=\kappa\sum_{\mathrm{event}=1}^{\kappa N} I_{\mathrm{event}}S_{\mathrm{event}}\sim\kappa\Delta I_{+},$$

which is less effective to control and eradicate the disease. Note that the original assumption that new cases are proportional to $S \cdot I$ relies on the idea that every individual interacts equally with all others. This assumption breaks down for very large event sizes, where individuals will not interact with more people if attendance increases.

**Appendix E: Exponential growth in the early pandemic: the fully susceptible limit**

This section provides an extended mathematical framework for mechanism M5. We present two complementary derivations of exponential growth: first, through the branching-process and generation-interval formalism, which yields the discrete-generation intuition used in the main text, and second, through the continuous SIR model, which provides an explicit exponential growth rate.

Exponential growth through branching and generation intervals. At the onset of a pandemic caused by a novel pathogen, the number of infected individuals is small relative to the population, so virtually every contact is with a susceptible individual. Under this condition, the spread of infection can be approximated by a Galton–Watson branching process: each infected individual independently gives rise to a random number of secondary infections with mean R0 before recovering [17].

Let Ik denote the expected number of infected individuals in generation k. By the branching-process recursion,

$$I_{k+1}=R_{0}\cdot I_{k},$$

Which gives the closed-form solution

$$I_{k}=I_{0}\cdot R_{0}^{k},$$

Where $I_{0}=I(0)$ is the initial number of infectious individuals. This is the fundamental discrete-generation exponential: the number of infections multiplies by $R_{0}$ with each successive generation interval $T_{g}$. If $R_{0} > 1$, case counts grow; if $R_{0} < 1$, the outbreak dies out.

The main-text statement that an intervention introduced $k$ generations later must contain an outbreak $R_{0}^{k}$ times larger follows directly from this formula. Table F1 illustrates the cumulative multiplicative burden $R_{0}^{k}$ for representative values of $R_{0}$ and $k$, showing how quickly the number of infected individuals increases even for modest reproduction numbers.

Table F1: Multiplicative increase in case burden at the moment of intervention as a function of delay (in discrete generations) and $R_{0}$

| $R_{0}$ | 1 generation late $(\times R_{0}^{1})$ | 2 generations late $(\times R_{0}^{2})$ | 3 generations late $(\times R_{0}^{3})$ |
| --- | --- | --- | --- |
| $1.5$ | $\times1.5$ | $\times2.3$ | $\times3.4$ |
| $2.0$ | $\times2.0$ | $\times4.0$ | $\times8.0$ |
| $2.5$ | $\times2.5$ | $\times6.3$ | $\times15.6$ |
| $3.0$ | $\times3.0$ | $\times9.0$ | $\times27$ |

**Exponential growth through the SIR model.** The branching-process argument above is formulated in discrete generations rather than continuous time. To obtain an explicit growth rate in continuous time and to connect $R_{0}^{k}$ to underlying epidemic parameters, we turn to the standard SIR model. We consider the standard SIR model with transmission rate $\beta$, recovery rate $\gamma$, and a population of size N partitioned into susceptible ($S$), infectious ($I$), and recovered ($R$) compartments:

$$\dot{S}=-\frac{\beta}{N}SI, \dot{I}=\frac{\beta}{N}SI-\gamma I, \dot{R}=\gamma I.$$

The basic reproduction number is $R_{0} =\frac{\beta}{\gamma}$, and the effective reproduction number at time t is $R_{\mathrm{eff}}\left( t \right)= R_{0} \cdot\frac{S\left( t \right)}{N}$.

At the onset of a pandemic caused by a novel pathogen, $S\left( 0 \right)\approx N$, so $\frac{S\left( t \right)}{N} \approx1$ for as long as the cumulative number of infections remains a negligible fraction of $N$. Under this approximation, the equation for $I$ decouples from $S$:

$$\dot{I}\approx\beta I-\gamma I= \gamma\left( R_{0}-1 \right)I.$$

This is a linear autonomous ODE with solution

$$I\left( t \right)=I_{0}\exp\left( \gamma\left( R_{0}-1 \right)t \right).$$

**Appendix F: The prevention paradox: network structure, information environment, and cultural context**

This section provides an extended discussion for mechanism M7. We examine how the architecture of modern social networks, the information environment they generate, and the cultural and institutional context in which individuals form beliefs together shape the severity and resolution of the prevention paradox.

**Why the paradox is structurally inevitable.** The core mismatch between subjective experience and epidemiological risk follows directly from the conditions under which mitigation is necessary. A pathogen with high severity and a finite healthcare system implies a very low prevalence threshold at which the system becomes overwhelmed; to stay below it, interventions must suppress transmission. Under effective control, the probability that any given individual personally encounters an active case in their immediate social circle is, by design, negligible. This absence of visible illness can, in turn, erode support for the very intervention that produced it—a self-undermining dynamic. It can only be resolved through communication that explicitly frames the absence of illness as the signature of a successful intervention rather than as evidence against it [18]. Institutional trust plays a key role here: where trust in public health authorities is high, populations are more likely to accept this and maintain compliance [19,20].

**How online networks decouple personal experience from local transmission dynamics.** In an offline social network under effective local control, one’s immediate social environment reflects, at least approximately, local transmission dynamics, and the low number of visible cases within it is a meaningful signal about local prevalence [21]. Modern online networks introduce a structurally distinct information channel that disrupts this correspondence. Content from regions with very different epidemiological situations enters the same feed as content from one’s own region, and its composition is governed by recommendation algorithms rather than by geography or social proximity [22,23]; at the same time, dramatic or emotionally engaging cases are systematically amplified relative to typical ones [24]. As a result, a successfully controlled local epidemic can register as either invisible or catastrophic depending on the composition of one’s feed, with neither impression reliably tied to actual local prevalence. A qualitatively different distortion arises from social ties that exist only online: regular contacts in gaming communities, topic-specific forums, or group messaging platforms are subjectively experienced as part of one’s social circle [25,26], yet transmit almost no embodied signals of illness. Parasocial relationships with high-reach content creators compound this further, as audiences weigh their reported experiences as they would those of an acquaintance, so a single creator’s narrative about illness—or its deliberate minimisation—can dominate perceived severity across an entire follower base out of all proportion to what a single individual’s experience can tell us about the population [27–30]. Both effects sever the link between one’s felt social environment and the local transmission dynamics on which the personal-network signal otherwise depends.

**Cultural and institutional context.** Cross-national analyses suggest that voluntary adherence to protective measures tends to be higher in societies whose cultural orientation places greater weight on collective obligations relative to individual autonomy [31–33], and that more fluid social structures are associated with faster epidemic growth, partly because effective contact networks are larger and less locally clustered [34–37]. In such settings, the personal-network signal is a weaker proxy for community prevalence even in the absence of online confounding. This interacts directly with the solidarity dynamics described in mechanism M9: in contexts of inclusive solidarity, the absence of visible illness can be collectively understood as a sign of shared success, reinforcing compliance; when solidarity fragments into narrower in-groups, the same absence is more readily reinterpreted as evidence that the threat was overstated [38–40]. Effective pandemic communication must therefore not only address the inferential gap between “I do not see illness around me” and “the intervention is working,” but do so in a manner calibrated to the network structure, media ecology, and cultural context in which that inference is made.

**Appendix G: Extended version of “Feedback between human behavior, spreading dynamics, and policy design”**

This section provides an extended discussion of mechanism M10. Epidemic and pandemic infectious disease outbreaks involve three major players with potentially conflicting aims: the pathogen, which tends to spread within a population; individuals, who aim to avoid becoming sick without restricting their daily activities; and policymakers, who aim to control the epidemic at minimal societal cost. These players interact to form a dynamical system with possibly complex dynamics, meaning that the actions of one player have non-trivial effects on the others. This has far-reaching consequences for how we build and interpret mathematical models for infectious diseases. A typical example is the adaptation of human behavior in response to current incidence of infection and hospitalizations and implemented interventions, which may cause interventions to counteract or complement one another. This adaptation is guided by peoples risk perception and their preferences regarding health-related behaviour, which can be assessed via surveys or other tools [41,42].

Importantly, the channels through which risk perception is formed have expanded with the growing dominance of social media in every communication and information exchange. Online platforms now rapidly diffuse both accurate health information and misinformation [43]. Algorithmic curation tends to reinforce pre-existing beliefs and foster echo chambers, in which users are predominantly exposed to like-minded content, making it harder to update risk perceptions in response to new information [44]. It is increasingly difficult for policy makers to compete with these social media influences in communicating on preventive measures, but social media sentiments have to be taken into account to predict adherence [45].

On the other hand, behaviour is determined by control measures implemented by health authorities. These can be varying in stringency and adherence to these measures depends on societal and cultural factors [1–3]. For example, the rapid spread of risk awareness and subsequent voluntary adoption of protective behaviors, such as mask wearing and hygiene measures, complemented the mandatory NPIs during the first COVID19 wave, helping to lower and postpone the epidemic peak [46]. The same complementarity led to short but strict lockdowns to mitigate more effectively than prolonged moderate measures, as they avoid the effects of pandemic fatigue [47, 48] and declining adherence [49]. Conversely, the rollout of COVID-19 vaccinations in 2021 led some vaccinated individuals to increase their contact rates to pre-pandemic levels, partially counteracting the mitigating effect of vaccination and potentially leading to higher short-term incidence [50–52]. A example of the latter is when a short term lifting of social distancing measures for vaccinated individuals (assessed with a digital Covid certificate) led to a large peak in incidence in the Netherlands in July 2021 [53]. These interactions between changing immune status of the population, peoples risk perception, adherence to measures, and policy decisions about interventions incur complex feedback loops. While we understand their mechanics, their specific dynamics can depend strongly on the societal setting, and are thus hard to predict [52]. These effects add to complexities of infection dynamics that are influenced by the interaction between host and pathogen, in particular by the hosts immune response and the built up and waning of immunity during and after epidemic waves [54]. Modeling the interplay among health opinions that spread through social influence, the pathogens spread, and policy responses is complex due to the heterogeneous and time-varying nature of these influences. Understanding the combined effect of such mechanisms requires first a deep understanding of each mechanism in isolation. Here, theoreticians need to strike a delicate balance between clarity and simplicity while acknowledging the complexity when translating the principles into real-world settings.

**References**

[1]	Haug N, Geyrhofer L, Londei A, Dervic E, Desvars-Larrive A, Loreto V, et al. Ranking the effectiveness of worldwide COVID-19 government interventions. Nature human behaviour. 2020;4(12):1303-12.

[2]	Brauner JM, Mindermann S, Sharma M, Johnston D, Salvatier J, Gavenčiak T, et al. Inferring the effectiveness of government interventions against COVID-19. Science. 2021;371(6531):eabd9338.

[3]	Hale T, Angrist N, Goldszmidt R, Kira B, Petherick A, Phillips T, et al. A global panel database of pandemic policies (Oxford COVID-19 Government Response Tracker). Nature Human Behaviour.

2021;5(4):529-38.

[4]	Snoeijer BT, Burger M, Sun S, Dobson RJ, Folarin AA. Measuring the effect of Non-Pharmaceutical Interventions (NPIs) on mobility during the COVID-19 pandemic using global mobility data. NPJ digital medicine. 2021;4(1):81.

[5]	Thomson S, Ip EC, Lee SF. International comparisons of COVID-19 case and mortality data and the effectiveness of non-pharmaceutical interventions: a plea for reconsideration. Journal of Biosocial Science. 2022;54(5):735-41.

[6]	Andrews MA, Bauch CT. The impacts of simultaneous disease intervention decisions on epidemic outcomes. Journal of theoretical biology. 2016;395:1-10.

[7]	Chin V, Ioannidis JP, Tanner MA, Cripps S. Effect estimates of COVID-19 non-pharmaceutical interventions are non-robust and highly model-dependent. Journal of Clinical Epidemiology. 2021;136:96-132.

[8]	Nouvellet P, Bhatia S, Cori A, Ainslie KE, Baguelin M, Bhatt S, et al. Reduction in mobility and COVID-19 transmission. Nature communications. 2021;12(1):1090.

[9]	Persson J, Parie JF, Feuerriegel S. Monitoring the COVID-19 epidemic with nationwide telecommunication data. Proceedings of the National Academy of Sciences. 2021;118(26):e2100664118.

[10]	Lucas Jr RE. Econometric policy evaluation: A critique. In: Carnegie-Rochester conference series on public policy. vol. 1. North-Holland; 1976. p. 19-46.

[11]	Funk S, Salathé M, Jansen VAA. Modelling the influence of human behaviour on the spread of infectious diseases: a review. Journal of the Royal Society Interface. 2010;7(50):1247-56.

[12]	Bedson J, Skrip LA, Pedi D, Abramowitz S, Carter S, Jalloh MF, et al. A review and agenda for integrated disease models including social and behavioural factors. Nature human behaviour. 2021;5(7):83446.

[13]	Bauch CT, Earn DJ. Vaccination and the theory of games. Proceedings of the National Academy of

Sciences. 2004;101(36):13391-4.

[14]	Wang Z, Bauch CT, Bhattacharyya S, d’Onofrio A, Manfredi P, Perc M, et al. Statistical physics of vaccination. Physics Reports. 2016;664:1-113.

[15]	Callaway B, SantAnna PH. Difference-in-differences with multiple time periods. Journal of econometrics. 2021;225(2):200-30.

[16]	Abadie A, Diamond A, Hainmueller J. Synthetic control methods for. Social connections and incentives in the workplace: Evidence from personnel data. 2010.

[17]	Watson HW, Galton F. On the probability of the extinction of families. The Journal of the Anthropological Institute of Great Britain and Ireland. 1875;4:138-44.

[18]	Messinger Cayetano S, Crandall L. Paradox of success and public perspective: COVID-19 and the perennial problem of prevention. Journal of Epidemiology and Community Health. 2020;74(8):679-9.

[19]	Bargain O, Aminjonov U. Trust and compliance to public health policies in times of COVID-19. Journal of public economics. 2020;192:104316.

[20]	Goldfinch S, Taplin R. A cross-sectional international study shows confidence in public health scientists predicts use of COVID-19 non-pharmaceutical interventions. BMC Public Health. 2022;22(1):662.

[21]	Giese H, Stok FM, Gaissmaier W, Wegwarth O. A social network perspective on social cues for COVID risk perception. Scientific Reports. 2025;15(1):15118.

[22]	Lerman K.	Information is not a virus, and other consequences of human cognitive limits.	Future

Internet. 2016;8(2):21.

[23]	Santos FP, Lelkes Y, Levin SA. Link recommendation algorithms and dynamics of polarization in online social networks. Proceedings of the National Academy of Sciences. 2021;118(50):e2102141118.

[24]	Caldarelli G, Artime O, Fischetti G, Guarino S, Nowak A, Saracco F, et al. The physics of news, rumors, and opinions. arXiv preprint arXiv:251015053. 2025.

[25]	Arroyo-Flores JG. Online–Offline Social Ties in Massive Multiplayer Online Games. 2019.

[26]	Prochnow T, Patterson MS, Hartnell L. Social support, depressive symptoms, and online gaming network communication. Mental Health and Social Inclusion. 2020;24(1):49-58.

[27]	Lee YH, Yuan CW, Wohn DY. How video streamers mental health disclosures affect viewers risk perceptions. Health Communication. 2021;36(14):1931-41.

[28]	Walter N, Cohen J, Nabi RL, Saucier CJ. Making it real: The role of parasocial relationships in enhancing perceived susceptibility and COVID-19 protective behavior. Media Psychology. 2022;25(4):601-18.

[29]	Harff DR, Bollen C, Schmuck D. Social Media Influencers’ Misinformation about COVID-19: A PreRegistered Multiple-Exposure Experiment. In: European Conference on Health Communication, Location: Online; 2021. .

[30]	Schmuck D, Harff D. Popular among distrustful youth? Social media influencers communication about COVID-19 and young peoples risk perceptions and vaccination intentions. Health Communication. 2024;39(12):2730-43.

[31]	Dinero R, Pieczonka A, Kmush BL. The association of cultural orientation with adherence to social distancing behaviors during the early COVID-19 pandemic in the United States: A cross-sectional survey. PLOS Global Public Health. 2022;2(8):e0000866.

[32]	Melton RJ, Sinclair RC. Culture and COVID-19: A global analysis of the successes of collectivist countries and the failures of individualistic countries. Available at SSRN 3954093. 2021.

[33]	Santana AP, Korn L, Betsch C, Böhm R. Lessons learned about willingness to adopt various protective measures during the early COVID-19 pandemic in three countries. PLoS One. 2022;17(3):e0265892.

[34]	Volz EM, Miller JC, Galvani A, Ancel Meyers L. Effects of heterogeneous and clustered contact patterns on infectious disease dynamics. PLoS computational biology. 2011;7(6):e1002042.

[35]	Miller JC. Spread of infectious disease through clustered populations. Journal of the Royal Society Interface. 2009;6(41):1121-34.

[36]	Petruzelli N, Kresge K. Analyzing Epidemic Thresholds on Dynamic Network Structures. In: 2021 Joint Mathematics Meetings (JMM). AMS;. .

[37]	Colman E, Colizza V, Hanks EM, Hughes DP, Bansal S. Social fluidity mobilizes contagion in human and animal populations. Elife. 2021;10:e62177.

[38]	Borinca I, Griffin SM, McMahon G, Maher P, Muldoon OT. Nudging (dis) trust in science: Exploring the interplay of social norms and scientific trust during public health crises. Journal of Applied Social Psychology. 2024;54(8):487-504.

[39]	Tsang PM, Prost A. Boundaries of solidarity: a meta-ethnography of mask use during past epidemics to inform SARS-CoV-2 suppression. BMJ global health. 2021;6(1).

[40]	Murray DR, Schaller M. Threat (s) and conformity deconstructed: Perceived threat of infectious disease and its implications for conformist attitudes and behavior. European Journal of Social Psychology. 2012;42(2):180-8.

[41]	Li N, Rambod B, Dukers-Muijrers N, Chevalier JM, Steijvers L, Kojan L, et al. General population preferences for health-related protective behaviors during infectious disease emergencies: a systematic review of conjoint-analysis studies. Social Science & Medicine. 2025:118721.

[42]	Michie S, Van Stralen MM, West R. The behaviour change wheel: a new method for characterising and designing behaviour change interventions. Implementation science. 2011;6(1):42.

[43]	Desai AN, Ruidera D, Steinbrink JM, Granwehr B, Lee DH. Misinformation and disinformation: the potential disadvantages of social media in infectious disease and how to combat them. Clinical Infectious Diseases. 2022;74(Supplement_3):e34-9.

[44]	Cinelli M, De Francisci Morales G, Galeazzi A, Quattrociocchi W, Starnini M. The echo chamber effect on social media. Proceedings of the national academy of sciences. 2021;118(9):e2023301118.

[45]	Williams SN, Dienes K, Jaheed J, Wardman JK, Petts J. Effectiveness of communications in enhancing adherence to public health behavioural interventions: a COVID-19 evidence review. Philosophical

Transactions of the Royal Society A: Mathematical, Physical and Engineering Sciences. 2023;381(2257).

[46]	Teslya A, Pham TM, Godijk NG, Kretzschmar ME, Bootsma MC, Rozhnova G. Impact of self-imposed prevention measures and short-term government-imposed social distancing on mitigating and delaying a COVID-19 epidemic: A modelling study. PLoS medicine. 2020;17(7):e1003166.

[47]	Petherick A, Goldszmidt R, Andrade EB, Furst R, Hale T, Pott A, et al. A worldwide assessment of changes in adherence to COVID-19 protective behaviours and hypothesized pandemic fatigue. Nature Human Behaviour. 2021;5(9):1145-60.

[48]	Priesemann V, Brinkmann MM, Ciesek S, Cuschieri S, Czypionka T, Giordano G, et al. Calling for pan-European commitment for rapid and sustained reduction in SARS-CoV-2 infections. The lancet. 2021;397(10269):92-3.

[49]	Brunekreef J, Teslya A, Buskens V, Nunner H, Kretzschmar M. Impact of adherence and stringency on the effectiveness of lockdown measures: a modelling study. PloS one. 2025;20(12):e0338818.

[50]	Teslya A, Rozhnova G, Pham TM, van Wees DA, Nunner H, Godijk NG, et al. The importance of sustained compliance with physical distancing during COVID-19 vaccination rollout. Communications medicine. 2022;2(1):146.

[51]	Bauer S, Contreras S, Dehning J, Linden M, Iftekhar E, Mohr SB, et al. Relaxing restrictions at the pace of vaccination increases freedom and guards against further COVID-19 waves. PLoS computational biology. 2021;17(9):e1009288.

[52]	Wagner J, Bauer S, Contreras S, Fleddermann L, Parlitz U, Priesemann V. Societal self-regulation induces complex infection dynamics and chaos. Physical Review Research. 2025;7(1):013308.

[53]	van der Veer BM, Gorgels KM, den Heijer CD, Hackert V, van Alphen LB, Savelkoul PH, et al. SARS-CoV-2 transmission dynamics in bars, restaurants, and nightclubs. Frontiers in microbiology. 2023;14:1183877.

[54]	Molla J, Farhang-Sardroodi S, Moyles IR, Heffernan JM. Pharmaceutical and non-pharmaceutical interventions for controlling the COVID-19 pandemic. Royal Society Open Science. 2023;10(12).
