# Supplementary material for: Mechanics of pandemics
Source: eClinicalMedicine. 2026 Jul 31;98:104101. doi: 10.1016/j.eclinm.2026.104101 (PMC13453475; doi:10.1016/j.eclinm.2026.104101)
Supplement: Supplementary file 2 [file mmc2.docx]

"The following translations in Spanish were submitted by the authors and we reproduce them as supplied. They have not been peer reviewed. Our editorial processes have only been applied to the original abstract in English, which should serve as reference for this manuscript

Resumen

El COVID-19 y pandemias anteriores han demostrado cómo las enfermedades pueden alterar, amenazar y transformar la vida cotidiana. Dado que los patógenos y las sociedades evolucionan continuamente, cada pandemia es diferente. Sin embargo, ciertos principios fundamentales de la transmisión de enfermedades parecen mantenerse constantes en los distintos brotes. Estos "mecanismos" se basan en leyes naturales o en la propia estructura de nuestra biología y sociedad. Este artículo recopila diez mecanismos fundamentales, seleccionados por un equipo multidisciplinario con experiencia en salud pública, medicina, epidemiología, ciencias políticas, matemáticas, física y psicología. Estos mecanismos, aunque tal vez subestimados, determinan en gran medida cómo se desarrollan y controlan las pandemias. Cuanto mejor logremos comprender estos mecanismos e integrar este conocimiento en nuestras sociedades, mejor podremos prepararnos para futuras pandemias y responder adecuadamente cuando ocurran.

The following translations in German were submitted by the authors and we reproduce them as supplied. They have not been peer reviewed. Our editorial processes have only been applied to the original abstract in English, which should serve as reference for this manuscript

# Zusammenfassung

COVID-19 und frühere Pandemien haben gezeigt, wie stark Infektionskrankheiten das tägliche Leben beeinträchtigen, bedrohen und verändern können. Da sich sowohl Gesellschaften als auch Krankheitserreger kontinuierlich weiterentwickeln, verläuft jede Pandemie unterschiedlich. Dennoch existieren grundlegende Prinzipien der Krankheitsausbreitung, die auf verschiedene Ausbrüche zutreffen. Diese „Mechanismen“ ergeben sich aus Naturgesetzen, der menschlichen Biologie sowie gesellschaftlichen Strukturen. In diesem Artikel werden 10 solcher Mechanismen vorgestellt, die von einer multidisziplinären Expertengruppe aus den Bereichen Public Health, Medizin, Epidemiologie, Politikwissenschaft, Mathematik, Physik und Psychologie zusammengetragen wurden. Diese möglicherweise unterschätzten Mechanismen prägen maßgeblich, wie sich Pandemien entwickeln und eingedämmt werden können. Je besser wir diese Mechanismen verstehen und das Wissen darüber in unseren Gesellschaften verankert, desto besser können wir uns auf zukünftige Pandemien vorbereiten und angemessen reagieren, wenn sie auftreten.
